# Comparison of Biological Activity of Field Isolates of *Steinernema feltiae* with a Commercial *S. feltiae* Biopesticide Product

**DOI:** 10.3390/insects12090816

**Published:** 2021-09-12

**Authors:** Joanna Matuska-Łyżwa, Paulina Żarnowiec, Wiesław Kaca

**Affiliations:** Department of Microbiology and Parasitology, Institute of Biology, Faculty of Natural Sciences, Jan Kochanowski University in Kielce, Uniwersytecka 7, 25-406 Kielce, Poland; paulina.zarnowiec@ujk.edu.pl (P.Ż.); wieslaw.kaca@ujk.edu.pl (W.K.)

**Keywords:** biological activity, environmental nematodes, morphometry, *Steinernema feltiae*

## Abstract

**Simple Summary:**

Entomopathogenic nematodes (Steinernematidae) are obligate insect parasites and are used for insect pest control, particularly on amenity grassland and in horticulture. Extensive surveys have been conducted across the globe to isolate locally adapted entomopathogenic nematodes species. The biological activity and morphology of three new isolates of *Steinernema feltiae* from Poland are described. New *S. feltiae* isolates from Poland showed close genetic similarity to other isolates of this species and exhibited a high reproductive rate and invasive capacity compared to the commercial biopesticide.

**Abstract:**

Insect trap studies were carried out to determine the presence of entomopathogenic nematodes (EPN) from the family Steinernematidae in the soils of Poland and to compare the biological activities of field nematode isolates with nematodes from commercial biopesticide. The fauna of these organisms in central Poland is poorly studied in both taxonomic and biological terms. Tilled soils representative of this region were sampled from cultivated fields. EPN were isolated from soil samples under laboratory conditions and identified using a key for species identification and molecular analysis. Basic morphometric parameters of infective juveniles and adult males of the first generation were determined. The research showed that males and infective juveniles *Steinernema feltiae* from Łoniów were the largest. The smallest infective juveniles were found in the isolate from Oblasy, and the smallest males in the isolate from Danków. In Poland, new field isolates showed close genetic similarity to other *S. feltiae* isolates. The research showed that the field isolates from Poland had greater infectivity and rate of reproduction compared with nematodes from the commercial biopesticide. The findings indicate the potential use of field *S. feltiae* isolates from Poland (iso1Lon, iso1Dan and iso1Obl) to develop new biopesticide products.

## 1. Introduction

The abundance and activity of soil fauna are largely dependent on the level of human activity within a given sampling area. Species diversity usually declines with increasing land development, resulting in a detrimental effect on biogeochemical cycles in ecosystems, disrupting the natural food webs and increasing the populations of plant pests. In crops, pest control may be particularly difficult in some areas due to, among other things, regulatory restrictions for applying plant protection chemicals. Therefore, the development of biological control methods to manage insect pests, using natural enemies, is an effective alternative strategy by which to achieve pest management, but without damage to the environment. 

The widely used biocontrol factors include: bacteria, viruses, fungi, entomophagous insects and entomopathogenic nematodes (EPN) [1,2,3,4,5,6,7,8,9]. With regard to EPN, it has been reported that local populations of nematodes can be more effective at reducing pests than the isolates recovered from other environments [10,11]. It has been demonstrated that the animal hosts of the EPN are positive factors, and it has been demonstrated that EPN can infect more than 200 insect species from several orders [12].

Research into new isolates of EPN indicates that they can differ greatly in terms of invasiveness and survival, and tolerance to various environmental factors [13,14,15,16]. This variability indicates that a screening program may identify new species and isolates that might be particularly useful for the biological control of insect crop pests. 

EPN are natural inhabitants of the soil environment [17,18,19,20,21]. Their development cycle is associated with those of the host insects, whose bodies are used by EPN for the multiplication and subsequent emergence of up to several generations of nematodes. In addition to the adult stage, there is an egg and four juvenile stages in the EPN lifecycle, with the third (infective) stage juvenile adapted to survive in the external environment outside the host insect. EPN are closely associated with bacteria of the genera *Xenorhabdus* (in *Steinernema* spp.) and *Photorhabdus* (in *Heterorhabditis* spp.), e.g., *X. bovienii* Akhurst, 1983, and *P. luminescens* Thomas et Poinar, 1979 [22], although it has been proven that these nematodes also form relationships with other symbiotic bacterial species [23,24]. The efficacy and biocontrol success of EPN can be enhanced through improved understanding of their biology and ecology. Many endogenous (microbiome) and environmental factors influence the survival of EPN and their transmission to the target species following their application as a biocontrol agent [23,25].

The biological activity of EPN depends on numerous environmental factors. For example, *S. feltiae* Filipjev, 1934, is a cold-adapted species, infecting hosts within the range 8–30 °C and reproducing at 10–25 °C [26,27,28]. Another important factor is soil moisture, which regulates nematode survival and motility [29,30,31]. Nematode infective juveniles find the most favorable conditions for invasion to be within the 25–40% soil moisture range. When the soil exceeds 50% moisture content, the biological activity of EPN declines [32]. The motility of nematode juveniles in the soil also depends on the soil structure. The greatest invasiveness was observed in sandy and sandy loamy soils, as the pore spaces in such environments provide optimal moisture and oxygenation conditions [17,32].

EPN populations are also influenced by biotic factors. The soil-dwelling infective juveniles are exposed to contacts with many organisms that share the same biotope, including bacteria, fungi and other nematodes. These organisms may reduce the EPN populations by food and territorial competency, or by their negative impact on EPN survival [33,34].

EPN are reported to be safe biological plant protection agents [35,36], and due to numerous advantages, such as tolerance of unfavorable conditions and the ability to infect several pest species, they are a good alternative to chemical plant protection products in agroecosystems where different phytophagous species may be present at the same time [37].

The broad host range of most EPN and their potential to reproduce and survive in the soil environment are the main advantages for the use of EPN for biological pest control. Research data suggest that selection of native isolates of nematodes offers more benefits than using commercial preparations that are based on one specific nematode isolate, which is not necessarily local to the agroecosystem in question [11]. Studies on the occurrence, biology and utilization of field EPN isolates have been conducted in many countries around the world [38,39,40,41,42]. Other studies have provided evidence that EPN show considerable variation in terms of biological activity, reproduction, host selection and tolerance to various environmental conditions. Thus, there is a need to gain a thorough knowledge on the natural populations of these potentially valuable organisms [43,44,45]. 

It is hypothesized that the activities of the isolates will be different, owing to their different origins and adaptations. 

It has been demonstrated that local populations of EPN can be more effective at reducing pests than the isolates recovered from other environments [11]. Studying the biology of new isolates from Poland may be beneficial for plant protection. A better understanding of the national entomopathogenic nematode fauna in Poland and their distribution is another important aspect of the research.

The aim of the work was achieved by identifying new environmental Polish isolates of *S. feltiae* with the use of morphometric and molecular methods, and by comparing the biological activities of isolated nematodes with the commercial EPN biopesticide tested on larvae of the greater wax moth (*Galleria mellonella* Lepidoptera, Pyralidae).

## 2. Materials and Methods

### 2.1. Soil Samples’ Collection and Nematodes’ Isolation

*Galleria mellonella* for bioassays were obtained from laboratory cultures held at the Department of Microbiology and Parasitology, Jan Kochanowski University, Poland. Insects were cultured at 25 °C on beeswax patches in ventilated polypropylene containers. The fourth-instar of *G. mellonella* larvae, with an average body weight of 140 mg, was used for all analyses.

**Figure A1:**
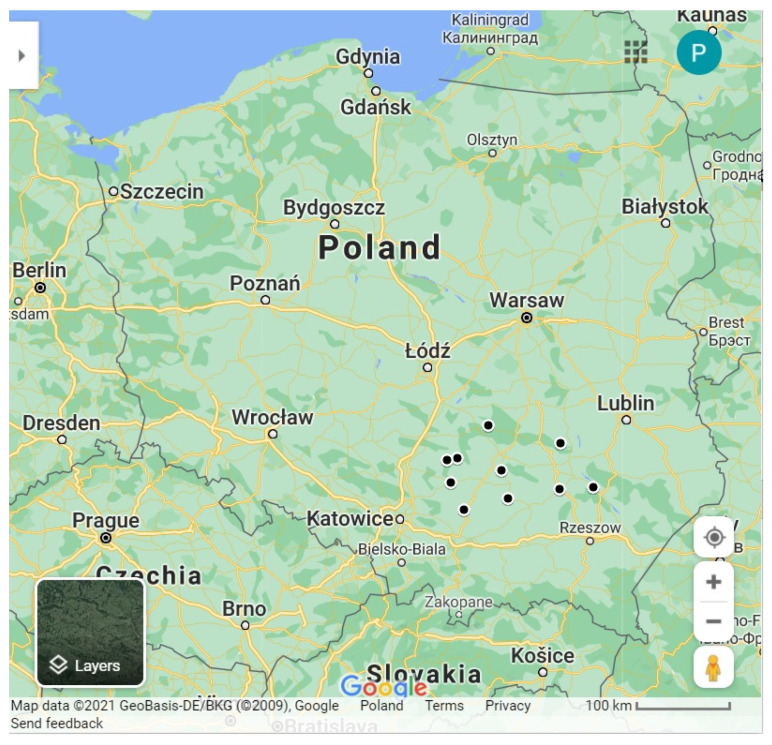
Location of sampling sites—dark circles (https://www.google.com/maps, accessed on 15 July 2021).

Ten organic farms on which wheat was grown were selected for the study (Appendix A, Table A1, Figure A1). For EPN isolation, soil samples were taken from sandy loam or loamy sand soils. At each farm, 50 soil samples were randomly collected to a depth of 25 cm from an area of 100 m^2^, using an Egner’s soil sampler. 

Under laboratory conditions, soil samples were mixed to obtain homogeneity and placed into 6 sterile 250 mL vessels. Field nematodes were isolated from soil samples using the insect trap method (*G. mellonella*) [46]. The samples were incubated in a thermostatically controlled temperature at 20 °C (POL-EKO Aparatura, Wodzisław Śląski, Poland) (optimal for the species [27]) and checked every 48 h over a 16-day period. 

Dead moth larvae, cleaned of soil particles and washed three times with distilled water, were transferred to migration sponges (modified White traps—patent No. PL 212617 B1 [47]), and fresh live larvae were introduced into the soil sample [46]. Nematodes’ migration on Petri dishes (Anumbra, Šumperk, Czech Republic) was also checked every 48 h, and newly emerging nematode larvae were collected into culture bottles and stored at 4 °C for further analysis [48].

### 2.2. Reproduction of the Collected EPN

Larvae of *G. mellonella* were infected with 50 EPN juveniles/insect on Petri dishes with filter paper, which were then stored in the incubation cabinet at 20 °C over a 5-day period. The cadavers of insects parasitized by nematodes were transferred into migration Petri dishes [47] (Anumbra, Šumperk, Czech Republic). The emerging juvenile nematodes were collected into tissue culture bottles, area 75 cm^2^ (Nunc EasyFlasks, Roskilde, Denmark), and stored at 4 °C. Field EPN isolates were subjected to morphometric and genetic analyses for species identification.

### 2.3. Identification of Nematode Species 

The preliminary nematode differentiation was based on the species identification key [49]. Morphometric and molecular analyses were used to identify the EPN species. Morphometric analysis of variables was carried out on invasive juveniles and adult males of the first generation [12,50,51]. For descriptive purposes, 25 specimens per variable were used. The following were measured: total body length, maximum body width, distance from anterior end to excretory pore, distance from anterior end to nerve ring, distance from anterior end to end of pharynx, tail length, anal body width and additionally, in males, spicule length and gubernaculum length.

### 2.4. Gene Sequencing

Genomic DNA was extracted using a Genomic Mini kit (A&A Biotechnology, Gdańsk, Poland). The internal transcribed spacer (ITS) region was amplified by PCR using forward and reverse primers SF18SL (5′GTACACACCGCCCGTCGCTGC3′) and SF18SR (5′AAATCCTAGTTAGTTTCTTTTCCTCCGC3′) [45]. 

The primers were used to determine the species. The PCR conditions were: 94 °C for 3 min and then 35 cycles (94 °C for 30 s, 66 °C for 30 s, 72 °C for 30 s), followed by an extension step at 72 °C for 5 min. After the last step, temperature was lowered to 4 °C, where the products were maintained until the purification step. The purification of the PCR product was performed using the PCR/DNA Clean-Up kit (A&A Biotechnology, Gdańsk, Poland) and sequenced in the laboratory CoreLab of the Medical University of Łódź, Poland. The purified PCR products were sequenced using the appropriate primers SF18SL and SF18SR. PCR sequencing was carried out in a 10 µL reaction volume, using Big Dye^®^ Terminator v1.1 (Applied Biosystems, South San Francisco, CA, USA). The PCR reaction was performed in a Gene Amp PCR System 9700 thermal cycler (Applied Biosystems, South San Francisco, CA, USA). Purification of the reaction products was performed using the BigDye XTerminator Purification Kit (Applied Biosystems, South San Francisco, CA, USA). Reaction conditions were set according to the manufacturer’s instructions. The reaction products were separated using a 3130xl Genetic Analyzer capillary sequencer (Applied Biosystems, South San Francisco, CA, USA). Sample analysis was performed using the DNA Baser tool for the DNA Sequence Assembler program (CoreLab, Medical University, Łódź, Poland).

### 2.5. Phylogenetic Analysis

The ITS gene sequence of isolates iso1Lon (from Łoniów), iso1Dan (Danków Duży) and iso1Obl (Oblasy), obtained in this study, were compared to the GenBank nucleotide sequences of other *S. feltiae* species using BLAST, available on the NCBI website (https://www.ncbi.nlm.nih.gov, accessed on 15 July 2021). The evolutionary relationship of the three isolates was inferred using the Neighbor-Joining (NJ) method with the phylogeny.fr (http://phylogeny.lirmm.fr/phylo_cgi/index.cgi, accessed on 15 July 2021) online software version 2021 for phylogenetic analysis [52]. 

### 2.6. Quantification of Biological Activity of EPN 

The *S. feltiae* isolate from the commercial Owinema biopesticide (Owiplant, Owińska, Poland) was used as a control to compare the biological activities of the three field isolates (iso1Lon, iso1Dan, iso1Obl). The insect mortality (percentage of insects killed due to any cause), infectivity (percentage of insects killed by nematodes), number of nematodes invading per insect, time to kill (days), time to first emergence of infective juveniles from infection (days) and number of infective juveniles emerging/insect were determined.

Larvae of *G. mellonella* were infected with 50 EPN juveniles/insect on Petri dishes with filter paper (50 IJs/0.1 mL), which were then stored in the incubation cabinet at 20 °C. For each sample (three field isolates and control sample), 60 larvae insects were used.

EPN infectivity was determined by dissection of insects 3 days after their death. Thirty insects from each study group (three field *S. feltiae* isolates and one control isolate) were dissected in Petri dishes. Using preparation needles, the body of one dead larvae was dissected into small sections and the number of nematodes inside the insect body was counted under a light microscope (number of nematodes invading per insect).

A similar approach was taken when analyzing the number of infective juveniles migrating from one insect. Insects that had been infected with the same number of nematodes (50 IJs/insect) were transferred on migration sponges, and after observing the emergence of the first nematode juveniles, the nematodes were collected. Five harvests (one every two days) were performed over a period of nine days. The experiment was repeated twice.

### 2.7. Statistical Analysis 

The Statistica version 13.3 software (statsoft.pl) was used for statistical analysis of data: morphometry and parameters of biological activity of nematodes. One-way analysis of variance (ANOVA) was performed for morphometric variables and the number of migrating infectious larvae. Mean and standard deviation were calculated for each morphometric variable in a given group. For biological activity parameters, Tukey’s test was used, with *p* < 0.05 used to differentiate homogeneous groups in multiple pairwise comparisons. Mean and standard deviation were calculated for the number of nematode juveniles migrating from one insect at successive harvests at two-day intervals and for the total number of migrating nematode juveniles from host insects from the different *S. feltiae* isolates.

## 3. Results

*Steinernema feltiae* was isolated from only three (Danków Duży (isolate iso1Dan), Łoniów (iso1Lon) and Oblasy (iso1Obl)) out of the ten regions sampled. *S. feltiae* was extracted from soils within a pH range of 5.2–5.4. The pH of the remaining soil samples was within the wider range of 5.2–5.6 (Table A1). 

**Figure 1:**
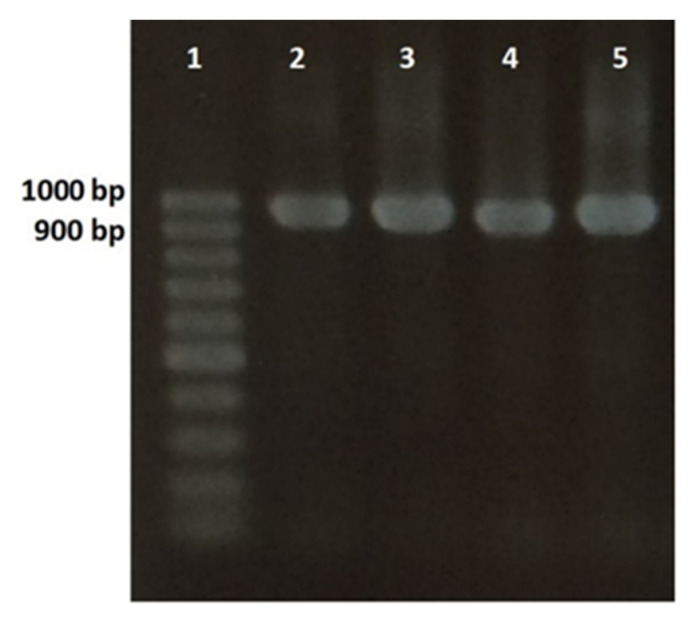
Agarose gel electrophoresis of internal transcribed spacer (ITS) PCR products from four *Steinernema feltiae* isolates: Lane 1: 100 bp DNA Ladder; Lane 2: PCR product of control sample (*S. feltiae* from biopreparation); Lane 3: PCR product of iso1Lon; Lane 4: PCR product of iso1Dan; Lane 5: PCR product of iso1Obl.

Molecular analysis and comparisons of the morphometric variables from the three EPN isolates with those specified in the key were performed, and in each case, the nematodes were identified as *S. feltiae* (Table 1 and Table 2, Figure 1). Invasive juveniles of the iso1Lon isolate were the largest of the isolates tested, whereas juveniles of the iso1Obl isolate were the smallest (Table 1). 

The males of the first-generation isolate from Łoniów were also larger than the males from Danków and Oblasy. In this generation, the iso1Dan isolate was the smallest. It was also observed that the length of the spicules was very similar for the isolates iso1Lon and iso1Obl (Table 2).

**Figure 2:**
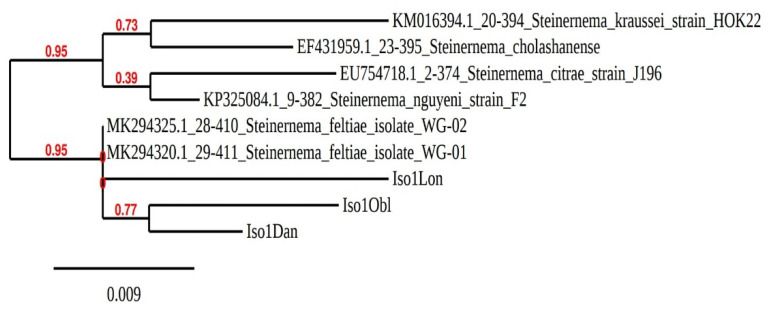
Phylogenetic relationships of three Poland EPN isolates based on analysis of internal transcribed spacers (ITS), using the Neighbor-Joining method.

Phylogenetic tree analysis, using the Neighbor-Joining method, based on ITS gene sequencing, revealed that the isolates from the three sites were very similar to other *S. feltiae* populations from Poland (Figure 2).

Insect mortality was shown to be 100% for the majority of the samples. In the sample infected with the iso1Obl isolate, the total mortality of the infected insects was lower, at 95%. 

With respect to the infectivity, only 2% of test insects in the control sample and 3% of insects in the samples infected with iso1Lon or iso1Obl isolates died for other reasons (dead insects were black and no nematodes were present at necropsy) (Table 3).

It was found that the number of nematodes invading per insect was significantly higher in the case of the iso1Lon isolate than with the other isolates (Table 3).

By analyzing the rate of development, it was shown that the control (Owinema) isolate and the iso1Dan isolate developed faster than the iso1Lon and iso1Obl isolates. A similar relationship was noted in the case of nematode reproduction, as the nematodes from the commercial preparation and the iso1Dan isolate started migrating within ten days of host infection, whereas the first progeny of the iso1Lon and iso1Obl isolates were observed migrating a day later (Table 3).

**Figure 3:**
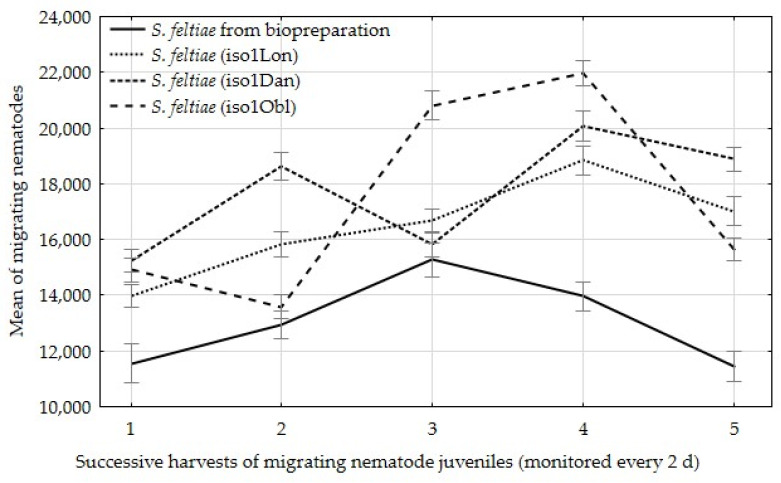
Mean of nematode juveniles migrating from one insect at successive harvests at 2-day intervals, ±standard deviation (SD).

When examining the number of migrating infective juveniles on successive days of migration, was observed that the emergence of infective juveniles from insect cadavers with field isolates reached a peak on the fourth day of emergence, whereas the nematodes from the control sample reached a peak on the third day of emergence (Figure 3).

**Figure 4:**
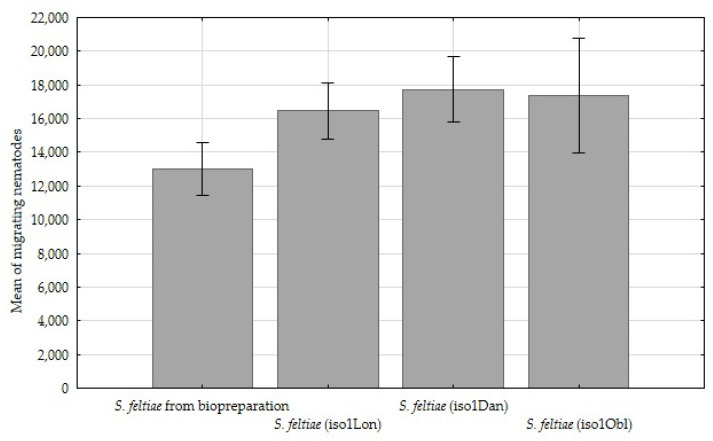
Comparison of mean of migrating nematode juveniles from host insects from the different *Steinernema feltiae* isolates, ±standard deviation (SD).

A comparison of the reproduction potentials revealed that the number of migrating infective juveniles was significantly (*p* < 0.05) higher, in the range of 5000, for all three environmental nematode isolates than for the control sample (Table 3, Figure 4).

The results indicate significant (*p* < 0.05) differences of invasion intensity and nematode reproduction between the field isolates of nematodes (iso1Lon, iso1Dan and iso1Obl) and the control group (Table 3, Figure 4).

## 4. Discussion

One of the topics that has attracted particular attention among entomonematologists is the study of the diversity and biological activity of EPN recovered from natural habitats [20,33,43,44,54,55,56,57,58,59,60,61,62]. About 93 species of *Steinernema* have been identified to date [55], of which 7 have been reported from the area of Poland [10,17,62].

The current study has shown that EPN recovered from cultivated soils in Poland were identified as *S. feltiae*. Morphological traits of the examined isolates matched the original description of the species, but the means of these dimensions were lower than in the original description [53]. Other studies indicate that the species has a global range, although it is most frequent under temperate climatic conditions [62,63].

Phylogenetic analysis using the Neighbor-Joining method based on ITS gene sequencing showed that new isolates (iso1Lon, iso1Dan and iso1Obl) formed a subcluster with other *S. feltiae* isolates.

The biological activity of the nematode isolates studied merits attention as it makes it possible to use local EPN isolates for the biological control of plant pests. The higher rate of survival and invasiveness by these field isolates, compared with the commercial biopesticidal isolate, would increase the quality and effectiveness of the biopreparation which could be prepared from one or more of these isolates.

Recent studies have provided evidence that EPN isolates originating from various sources differ in their biological activity [10,43,44,64], while other investigations found that the local populations of EPN were better adapted to native conditions and displayed a greater biological activity under such conditions than that achieved by isolates originating elsewhere [11]. Similar results were noted in this current study. The field nematode isolates were a little slower in developing than the commercial isolate, but penetrated insect bodies more readily than did the nematodes derived from commercial biopreparation.

EPN vary in their development rate [10,65] and may also be influenced by differences in their microbial association [25]. Some data from the literature suggest that EPN may kill insects within 24–48 h [61]. Other research has shown that to kill the insect, the nematodes needed more time [66]. In the current study, nematodes from the commercial biopesticide and the isolate from Danków Duży killed the insect host within two days, while the nematodes from Łoniów and Oblasy needed two to three days to achieve the same effect.

In this study, in terms of nematode biological activity (invasion extensiveness and intensity), the results were approximate to the results found by other authors from other countries [65,67]. The significantly greater invasion intensity in the field isolates than in the control isolate appears to reflect the greater invasiveness reported of field EPN isolates [44].

Other parameters of EPN bioactivity include their reproductive potential and their capacity to migrate from the host body. In the present study, it was demonstrated that nematodes isolated from the field needed more time to start migrating than did nematodes derived from the commercial biopesticide. A comparison of nematode reproductivity throughout the early days of migration showed that nematode isolates recovered from the field had a higher reproduction rate than did nematodes from the commercial biopesticide. Similar findings have been reported for these organisms in other studies [10,65,68,69].

The field *S. feltiae* nematodes were isolated from sandy soil, one of the dominant agricultural soil types in Poland, as well as throughout northwest Europe (France, Germany, Austria, Scandinavia, Great Britain). Therefore, the production of biopesticides from field *S. feltiae* isolates could have a wide economic impact for the protection of crops in Poland, as well as in other European countries.

The results obtained from this research confirm that field isolates are characterized by greater biological activity (higher invasion intensity and greater number of juvenile offspring from one host insect) than *S. feltiae* isolates originating from the Owinema biopreparation.

Additionally, this study supports the potential use of field entomopathogenic *S. feltiae* isolates for the production of effective biopreparations for crop protection.

## 5. Conclusions

*Steinernema feltiae* was reported in cultivated soils in Poland. New isolates showed close genetic and morphological similarity to other *S. feltiae* isolates. The field nematode isolates were characterized by a similar insecticidal effectiveness, which was confirmed by comparable infectivity. Field nematode isolates exhibited a high reproduction rate and number of nematodes invading per insect capacity compared with *S. feltiae* from commercial biopesticide.

## Figures and Tables

**Table 1:** Morphometric variables (mean ± SD) of infective juveniles of *Steinernema feltiae* from Poland. Variables (in µm) are analyzed by one-way ANOVA with correspondent F statistic, with degrees of freedom (df) and *p*-value.

Character	Infective Juvenile					
	Control (*S. feltiae* from Biopreparation)	*S. feltiae* (iso1Lon)	*S. feltiae* (iso1Dan)	*S. feltiae* (iso1Obl)	*S. feltiae* [53]	F (df_1_, df_2_), *p*
n	25	25	25	25	20	
L	861.2 ± 47	875 ± 54	816 ± 46	749 ± 54	879 ± 49	F = 29.9666 (3, 96), *p* < 0.00001
	(760–950)	(768–927)	(720–890)	(640–850)	(766–928)	
W	27 ± 1.7	28 ± 1.9	28 ± 2	26 ± 1.4	29 ± 1.9	F = 9.5558 (3, 96), *p* = 0.00001
	(25–32)	(24–31)	(25–32)	(23–29)	(26–32)	
EP	59 ± 1.9	61 ± 2.5	58 ± 1.5	56 ± 1.9	63 ± 2.3	F = 26.0012 (3, 96), *p* < 0.00001
	(57–63)	(57–65)	(56–61)	(53–59)	(58–67)	
NR	111 ± 3.2	112 ± 2.8	111 ± 1.7	106 ± 2	113 ± 5.1	F = 29.1955 (3, 96), *p* < 0.00001
	(107–116)	(108–116)	(108–115)	(102–110)	(108–117)	
ES	135 ± 3.6	135 ± 3.4	134 ± 3	128 ± 3.4	136 ± 3.5	F = 24.7273 (3, 96), *p* < 0.00001
	(130–142)	(128–141)	(127–141)	(120–136)	(130–143)	
T	82 ± 1.7	83 ± 1.9	82 ± 2.4	79 ± 2	86 ± 2.6	F = 14.944 (3, 96), *p* < 0.00001
	(80–86)	(79–87)	(77–87)	(75–83)	(81–89)	
ABW	16 ± 1.1	17 ± 0.7	16 ± 0.8	15 ± 0.8	18 ± 0.8	F = 20.6043 (3, 96), *p* < 0.00001
	(15–18)	(15–18)	(14–18)	(13–17)	(16–19)	
a	31.3	31.2	28.9	29	30	-
b	6.4	6.5	6.1	5.9	6.4	-
c	10.4	10.5	9.9	9.4	10	-
D%	43.8	45.1	43.4	43.7	46	-
E%	71.8	73.4	71	70.4	74	-

n = number of specimens, L = total body length, W = maximum body width, EP = distance from anterior end to excretory pore, NR = distance from anterior end to nerve ring, ES = distance from anterior end to end of pharynx, T = tail length, ABW = anal body width, a = L/W, b = L/ES, c = L/T, D% = (EP/ES) × 100, E% = (EP/T) × 100, - = not available. [53] refers to Nguyen et al., 2006. Numbers in brackets denote the range of the variable in question.

**Table 2:** Morphometric variables (mean ± SD) of first-generation adult males of *Steinernema feltiae* from Poland. Variables (in µm) are analyzed by one-way ANOVA with correspondent F statistic, with degrees of freedom and *p*-value.

Character	Male First Generation					
	Control (*S. feltiae* from Biopreparation)	*S. feltiae* (iso1Lon)	*S. feltiae* (iso1Dan)	*S. feltiae* (iso1Obl)	*S. feltiae* [53]	F (df_1_, df_2_), *p*
n	25	25	25	25	20	
L	1559 ± 93	1582 ± 91	1513 ± 88	1546 ± 96	1612 ± 88	F = 2.3369 (3, 96), *p* = 0.07851
	(1360–1720)	(1410–1795)	(1370–1690)	(1330–1769)	(1414–1817)	
W	134 ± 10.7	138 ± 9	124 ± 10	133 ± 10	140 ± 10	F = 8.759 (3, 96), *p* = 0.00003
	(118–157)	(118–160)	(105–140)	(108–158)	(121–162)	
EP	113 ± 3.6	115 ± 3.2	110 ± 2.9	112 ± 2.4	115 ± 3.4	F = 12.2043 (3, 96), *p* < 0.00001
	(108–121)	(109–123)	(105–115)	(108–116)	(110–126)	
ES	169 ± 4.5	169 ± 3.5	163 ± 4	166 ± 4.8	170 ± 3.4	F = 12.0016 (3, 96), *p* < 0.00001
	(161–176)	(163–177)	(155–170)	(159–175)	(164–180)	
T	36 ± 1.6	37 ± 1.4	35 ± 1.3	36 ± 1.4	39 ± 1.2	F = 6.3343 (3, 96), *p* = 0.00058
	(34–41)	(35–41)	(33–39)	(34–40)	(37–43)	
ABW	44 ± 2.4	47 ± 2.3	42 ± 2.4	43 ± 2.1	48 ± 1.7	F = 20.1168 (3, 96), *p* < 0.00001
	(42–51)	(42–52)	(36–48)	(37–49)	(43–53)	
SL	65 ± 1.7	64 ± 1.7	63 ± 1.8	64 ± 1.6	66 ± 1.5	F = 6.0791 (3, 96), *p* = 0.00078
	(61–68)	(61–68)	(58–67)	(60–66)	(62–68)	
GL	51 ± 2.2	51 ± 2.2	47 ± 2.3	49 ± 2.1	52 ± 1.9	F = 13.4403 (3, 96), *p* < 0.00001
	(47–57)	(46–55)	(42–52)	(44–54)	(48–56)	
a	11.67	11.43	12.22	11.63	11.5	-
b	9.3	9.3	9.3	9.3	9.5	-
c	43.1	42.5	42.7	42.8	41.3	-
D%	67	68	67.6	67.5	68	-
E%	-	-	-	-	-	-

n = number of specimens, L = total body length, W = maximum body width, EP = distance from anterior end to excretory pore, ES = distance from anterior end to end of pharynx, T = tail length, ABW = anal body width, SL = spicule length, GL = gubernaculum length, a = L/W, b = L/ES, c = L/T, D% = (EP/ES) × 100, E% = (EP/T) × 100, - = not available. [53] refers to Nguyen et al., 2006. Numbers in brackets denote the range of the variable in question.

**Table 3:** Biological activity of *Steinernema feltiae* isolates (mean values).

Parameter	Control *S. feltiae* (from Biopreparation)	*S. feltiae* (iso1Lon)	*S. feltiae* (iso1Dan)	*S. feltiae* (iso1Obl)
Insect mortality (%)	100 ^a^	100 ^a^	100 ^a^	95 ^b^
Infectivity (%)	98 ^a^	97 ^a^	100 ^a^	92 ^a^
Number of nematodes invading per insect (no. of ind.)	10 ^a^	27 ^c^	12 ^b^	12 ^b^
Time to kill insects (days)	2 ^a^	3 ^a^	2 ^a^	3 ^a^
Time from nematode infection to emergence of infective juveniles (days)	10 ^a^	11 ^a^	10 ^a^	11 ^a^
Number of infective juveniles emerging per insect (no. of ind.)	65,150 ^a^	82,320 ^b^	88,640 ^b^	86,910 ^b^

^a,b,c^—For a particular variable, any two isolates with a different superscript letter are significantly different (*p* < 0.05), using the Tukey test.

## Data Availability

Dataset available upon request to the corresponding authors.

## Appendix A

### Characteristics of the Areas of S. feltiae Isolation

The isolation of local populations of *S. feltiae* was carried out in the summer of 2019 on arable soils on which wheat had been grown. Soils were of the type representative of the arable soils in Poland (acidic sandy loam and loamy sand soils) and were typical of a temperate climate (Table A1, Figure A1).

**Table A1:** Characteristics of soil samples tested for entomopathogenic nematodes.

Locality	Latitude (N)	Longitude (E)	Soil Type	pH
Bałtów	51°01′02.6″	21°32′10.7″	loamy sand	5.2
Busko-Zdrój	50°28′06.5″	20°41′47.3″	sandy loam	5.2
Danków Duży	50°52′26.5″	19°55′29.6″	sandy loam	5.2
Końskie	51°11′38.9″	20°23′17.3″	loamy sand	5.4
Łoniów	50°33′31.1″	21°31′37.2″	sandy loam	5.2
Miechów	50°21′04.5″	20°03′07.4″	loamy sand	5.6
Morawica	50°44′48.4″	20°36′52.5″	loamy sand	5.4
Oblasy	50°50′20.1″	19°46′31.5″	sandy loam	5.4
Stalowa Wola	50°36′15.2″	22°02′32.3″	loamy sand	5.2
Szczekociny	50°37′14.5″	19°48′01.1″	loamy sand	5.6
